# Plant based production of myoglobin - a novel source of the muscle heme-protein

**DOI:** 10.1038/s41598-020-57565-y

**Published:** 2020-01-22

**Authors:** Magnus L. R. Carlsson, Selvaraju Kanagarajan, Leif Bülow, Li-Hua Zhu

**Affiliations:** 1 Department of Plant Breeding, Swedish University of Agricultural Sciences, Box 101, 230 53 Alnarp, Sweden; 2 Division of Pure and Applied Biochemistry, Department of Chemistry, Lund University, Box 124, 221 00 Lund, Sweden

**Keywords:** Plant biotechnology, Biotechnology, Molecular biology, Plant sciences

## Abstract

Myoglobin is a heme-protein in the muscle of vertebrates with important functions in the oxygenation of tissues and as a regulator in nitric oxide signaling. Myoglobin from many species is also an important nutritional source of bioavailable iron. In this study, we have successfully produced human myoglobin in the leaves of *Nicotiana benthamiana* by transient expression using a viral vector delivered by *Agrobacterium tumefaciens*. Analyses confirmed that heme was incorporated and the protein was functional, with observed properties consistent with those of native myoglobins. A relatively high degree of purity could be achieved with low cost methods. The results show the high potential of plants as a production platform for heme proteins, a group of proteins of interest for iron nutrition applications and possible future pharmaceutical development.

## Introduction

As a heme-protein in the muscle tissues of vertebrates, myoglobin (Mb) has been described as an oxygen binding protein, which facilitates diffusion of oxygen to the mitochondria^1^. Another important role attributed to Mb is regulation of nitric oxide signaling^2^. Mb was the first protein to have its structure solved^3^ and its properties and functions have been investigated in detail^4–6^.

Apart from their vital physiological roles in the transport and storage of oxygen, the heme-proteins of Mb and hemoglobin (Hb) are important nutritional sources of bioavailable iron. While non-heme iron is typically more plentiful in the diet, the organic heme-iron is more easily taken up by the body, and provides a significant part of the bioavailable iron in non-vegetarian diets^7^. Iron is an essential micronutrient, with a reported estimated daily requirement of 6 mg for adult men (18 years and above) and 8.1 mg for adult, premenopausal women of 19–50 years old^8^. Iron deficiency and resulting anemia have been described as a worldwide health concern, with particular prevalence in many developing countries^9,10^. Iron biofortification of crop species, using plant breeding, genetic engineering and other strategies, is one option that is being explored to help remedy this problem^11,12^. The ethical, environmental and economic concerns surrounding meat eating^13,14^ further motivate the need for alternative products capable of filling its nutritional role. Production of heme-proteins, such as Mb, in plants could be one alternative, by providing an important iron source of plant origin, something of particular importance for vegetarians. Very recently, another type of heme-protein produced in yeast using recombinant technology has been used as an additive in commercial food products, in this case the manufacturers primarily motivates this by a wish to mimic the flavor of meat in their vegetarian food^15^. This helps to illustrate how added heme-protein can bring value to plant based food and its consumers.

Another reason for interest in the heterologous expression of heme-proteins is for the development of oxygen therapeutics, a class of protein pharmaceuticals being developed with the aim of providing an alternative to donated blood in clinical situations, and often relies on a chemically modified Hb^16,17^. Many oxygen therapeutics concepts rely on cell-free Hb from animal sources or excess donated blood^16^. However, the use of recombinant technology for the production of oxygen therapeutics may provide alternative sources for cost effective and sustainable production of these protein pharmaceuticals. *E*. *coli* and yeast have been shown to express Hb with good yields and have been considered as suitable production platforms^18,19^. A previous study has also shown stable expression of human Hb in transgenic plant tissue with very low yield^20^. Use of recombinant technology could allow for the production of a larger selection of heme proteins, such as fetal Hb^21^, and for the control of their properties through genetic modifications^19,22,23^, while also minimizing the risk of transmissible diseases. As a simpler monomeric heme protein, Mb produced using heterologous expression techniques could provide a valuable resource in the development of oxygen therapeutics, and help show the feasibility of producing similar heme-proteins in plants for potential pharmaceutical applications.

To the best of our knowledge, expression of Mb in plants has not been previously reported. The scalable and sustainable nature of plant cultivation could make it a valuable option for heme protein production. Moreover, plants have a particular advantage for the production of Mb as heme is produced in plants and shares most of its synthesis pathway with chlorophyll^24^. The precursors to heme synthesis could therefore be expected to be available in quantity in plant tissues, especially in green leaves. In comparison, the supply of heme during bacterial or yeast expression can be an issue, and may require workarounds, such as addition of heme or its precursors, or genetic engineering strategies^19^. For common bacterial expression systems, i.e. *E*. *coli*, immunogenic lipopolysaccharides complicates the production of safe oxygen therapeutics and adds to the purification burden^18^, while the lack of these compounds in plants could be an additional advantage for this expression system. Furthermore, for applications such as human nutrition, ethical, environmental and other concerns might also make plant-based products more attractive to consumers, compared to those produced using microbial- or animal-based production systems.

Various techniques have been developed for expression of heterologous proteins in plants. These can be based on stable transformation where a piece of external DNA is integrated into the plant genome, or transient, through which no external DNA is inserted into the plant genome^25–27^. It has been shown that transient plant expression systems, in particular when combining *Agrobacterium* with viral vectors, are capable of a high level of expression of heterologous protein^28–30^.

The aim of this study was to investigate the possibility of producing Mb in plants. For this purpose, the human *Mb* gene was selected and cloned into a viral vector, which was then transferred into the leaf cells of *Nicotiana benthamiana* using *Agrobacterium tumefaciens* for transient expression. The results showed that the human Mb protein was successfully expressed in the leaves. Further analyses confirmed that the purified protein was functional and displayed physicochemical properties very similar to native Mbs.

## Materials and Methods

### Plant material

Seeds of *N*. *benthamiana* were sown in pots and grown for 2 weeks, then transplanted and grown individually in 2 L pots. The plants were grown in a controlled climate chamber in the biotron at the Swedish University of Agricultural Sciences (SLU), Alnarp. The climate conditions were 18 h light at 250 µmol m^−1^ s^−1^ with the temperature of 25 °C (day) and 6 h at 20 °C (night) and 60% relative humidity. Agroinfiltration or agrospray application of *Agrobacterium* suspension was carried out when plants were 5–6 weeks old.

### Construct design and gene synthesis

The sequence of the human *Mb* gene, was acquired from the Uniprot database (accession number of P02144)^31^. A leading Kozak consensus sequence and flanking restriction sites were added to the gene sequence. The sequence was codon optimized for expression in *N*. *benthamiana* and synthesized by the Thermo Fisher GeneArt Service (Waltham, MA, USA). Two versions of the *Mb* gene were designed; one intended for accumulation of the protein in the cytosol and the other intended for accumulation in the chloroplast. The latter was fused to the *N*. *tabacum* rubisco small subunit chloroplast targeting peptide (Uniprot database, accession number P69249^31^) for the chloroplast localization.

### Preparation of transient expression vectors and molecular cloning

The tobacco mosaic virus based pJL-TRBO vector^29^ was used in this study. The pJL-TRBO vector and the synthesized vectors, containing the *Mb* sequences, were digested with *Pac*I and *Xma*JI FastDigest restriction enzymes (Thermo Fisher Scientific). The *Mb* fragments were isolated using an agarose gel and purified using a gel extraction kit (Thermo Fisher Scientific). The *Mb* sequences were then cloned into the pJL-TRBO vector and transformed into competent cells of *E*. *coli* (Takara Bio, Kusatsu, Japan) following the manufacturers’ protocol. The bacteria were then cultured on the Luria-Bertani (LB) medium with kanamycin for selection and the presence of the ligated vectors were confirmed by colony PCR using vector specific primers. The PCR positive plasmids were further confirmed by sequencing by Eurofins Genomics (Ebersberg, Germany) and then transformed into competent cells of *A*. *tumefaciens* GV3101:pMP90 by electroporation for further use.

### Agroinfiltration and agrospray

The preparation of *Agrobacterium* inoculation suspensions for agroinfiltration or agrospray were carried out essentially according to the description by Lindbo^29^. Prior to its application to the plants, the *Agrobacterium* suspension containing the pJL-TRBO vector with the *Mb* gene and the *Agrobacterium* suspension containing the pJL3-p19 vector were mixed in a 2:1 ratio.

For agroinfiltration, the inoculation solution was injected into the abaxial side of the leaves using a syringe. Agroinfiltrated leaves were harvested 7 days after infiltration (DAI), and frozen at −80 °C. For agrospray, the inoculation solution was diluted up to 20x in 10 mM MES pH 5.7, 10 mM MgCl_2_ with addition of Silwet L-77 to 0.05% immediately prior to spraying the plants. The inoculation solution was applied to both sides of the leaves using a handheld spray. Agrosprayed leaves were harvested at 9–14 DAI and frozen at −80 °C. For the production of the purified material 20x dilution and 14 DAI harvest were used.

### Protein extraction and purification

The harvested leaves were grinded into fine powder in a RM200 mortar grinder (Retsch, Haan, Germany), precooled with liquid nitrogen. Leaf midribs were removed from the harvested leaves to facilitate grinding, either at harvest or immediately prior to grinding.

The Mb protein was extracted from the leaf powders by addition of 2–3× (V/W) of the extraction buffer (20 mM sodium phosphate buffer pH 6.3, 5 mM dithiorethiol (DTT), 5% W/V polyvinylpolypyrrolidone (PVPP), 1 mM ethylenediaminetetraacetic acid (EDTA), 0.1% plant protease inhibitor cocktail (Sigma-Aldrich, St. Louis, USA)). The extract was then centrifuged at ~12000 RCF for 20 min at 4 °C and filtered using miracloth or nylon cell strainers.

Following extraction, the sample was bubbled using carbon monoxide (CO) and heated in a 60 °C water bath for at least 10 min. The precipitated proteins were removed by centrifugation at 4 °C, followed by filtration using 0.2 µm sterile filters. For initial characterization, shorter heating times were used to evaluate the heat stability of the protein. The Mb extract was concentrated to a minimal volume (~20–50 ml) using a Vivaflow 50 R (10 kDa MWCO) and brought to pH 8.5 by addition of 200 mM Tris-HCl buffer pH 9.0, 5 mM DTT, 1 mM EDTA, bubbled with CO. The extract was then brought to a final concentration of 2.28 M ammonium sulfate (approximately 60% saturation at 4 °C), by dropwise addition of ammonium sulfate solution (50 mM Tris-HCl pH 8.5, 5 mM DTT, 1 mM EDTA, 3.8 M ammonium sulfate) while under stirring in an ice bath. The extract was then buffer changed using a Vivaflow 50 R (10 kDa MWCO), by serial dilution with buffer solution (5 mM Tris-HCl pH 8.5 (4 °C), 1 mM DTT), bubbled with CO.

Following the buffer exchange, Mb was purified by anion exchange chromatography using 5 ml Hitrap HP Q-Sepharose columns (GE life sciences), with up to three columns connected in series, operated by a Biologic LP Chromatography System (Bio-Rad, Hercules, CA, USA). Columns and buffers were kept in an ice bath during operation. The columns were equilibrated with 5 mM Tris-HCl buffer pH 8.5 (4 °C). Following sample application, the columns were washed with ~5 column volumes of the equilibration buffer and an elution gradient of 0–100 mM NaCl over 10 to 20 column volumes was applied to the column. Mb fractions were then collected based on visible color and chromatogram data. Vivaspin 15 R concentrators (5000 MWCO) or Sephadex G25 PD10 columns were used to remove remaining salts and for general buffer change as required for analysis. For storage, the purified Mb was bubbled with CO and flash frozen by submersion in liquid nitrogen. All steps of protein extraction and purification were carried out at 4 °C or on ice whenever possible.

### Protein analysis

#### SDS-PAGE and Western Blot

SDS-PAGE was performed to determine the purity and approximate molecular weight of the Mb protein. Sample reaction mixtures of 20 µl consisting of 5 µl bolt sample buffer (Thermo Fisher Scientific), 2 µl bolt reducing agent (Thermo Fisher Scientific), 1–13 µl Mb protein sample and dH_2_O were heated for 10 min at 70 °C. The samples were loaded in precast NuPAGE 4–12% Bis-Tris protein gels (Thermo Fisher Scientific) and were run for 45 min at 145 V and 400 mA. The gels were then washed for 5 min (3 x) in Millipore water and stained with SimplyBlue SafeStain (Thermo Fisher Scientific) for at least 1 h, then destained in Millipore water. SeeBlue Plus2 (Thermo Fisher Scientific) was used as a protein standard.

For Western blot analysis, the purified protein sample was first run on SDS-PAGE gels as stated above. After washing the gels in Millipore water, the proteins were transferred to a membrane using an iblot 2 PVDF mini stack and the iblot 2 system (Thermo Fisher Scientific) using the default program. The membranes were retrieved and washed twice in Millipore water, and then treated with superblock blocking buffer for 30 min under shaking. Thereafter the membranes were treated with the primary anti-Mb antibody solution (monoclonal anti-Mb,Abcam ab77232 (Abcam, Cambridge, United Kingdom), 5000x diluted) for 30 min, followed by washing in PBS-Tween 20 (Thermo Fisher Scientific) for 1 min under shaking and three consecutive washes of 5 min each. The membrane was treated with the secondary antibody (HRP-conjugated goat anti rabbit antibody, A16096 (Thermo Fisher Scientific)) for 30 min under shaking, then washed as before. Finally, the membranes were washed with Millipore water for 2 min, and treated with Novex ECL chemiluminescent substrate reagent mixture (Thermo Fisher Scientific). A ChemiDoc MP imaging system (Bio-Rad) was used to capture the images.

#### Mb quantification

The purified Mb protein was quantified using a Multiskan GO Spectrophotometer (Thermo Fisher Scientific) and the millimolar extinction coefficients of 14.9 for 542 nm and 12.8 for 580 nm. These values were obtained by converting the units of the extinction coefficients reported by de Duve^32^. Observations of the relative absorbance of oxy-Mb and carboxy-Mb were also used to estimate the concentration of oxy-Mb using these extinction coefficients. A Samsung galaxy S8 was used to capture images in order to record the color difference between the extracts of agroinfiltrated and untreated tobacco.

#### Absorbance spectra and ligand binding

To confirm that the purified Mb protein was functional, the sample stored at −80 °C under CO was thawed and buffer changed to 20 mM phosphate buffer pH 7.0 using a Sephadex G25 PD10 column (GE Healthcare, Chicago, IL, USA). The collected fractions were then combined and briefly bubbled with air under strong light. The sample was then aliquoted in order to prepare the different Mb-ligand complexes. An oxy-Mb spectrum was measured without further treatment of the sample. A carboxy-Mb sample was bubbled with CO and measured under CO in a sealed cuvette, with or without adding a few grains of sodium dithionite immediately before the measurement. A deoxy-Mb spectrum was recorded after briefly bubbling the sample with N_2_ from a Nitrovap generator (Parker Hannifin, Cleveland, Ohio, USA), followed by addition of a few grains of sodium dithionite. The sample was then sealed in a cuvette during the measurement. Met-Mb was generated by adding a 1.5x molar excess of potassium ferricyanide, based on the concentration calculated from the carboxy-Mb spectrum. Each sample was analyzed on a Multiskan GO Spectrophotometer (Thermo Fisher Scientific) in the range 250–700 nm with 1 nm steps at room temperature in quartz cuvettes with a 1 cm path length. Mb absorption spectra collected before or after the chromatographic purification were also used to estimate the purity by calculating the ratio between the Mb β-peak absorbance maximum at around 540 nm and the absorbance maximum at around 280 nm, for this ratio, a higher value would mean higher purity.

#### Structural and stability tests

The purified Mb protein produced in *N*. *benthamiana* was sent to Alphalyse (Odense, Denmark) for molecular mass determination using LC-ESI-MS. The CD-spectra of the 250–190 nm region were recorded using a Jasco J-815 CD spectrometer (Jasco, Hachioji, Japan). A sample of ~0.12 mg/ml Mb in 100 mM phosphate buffer was measured in a 1 mm cuvette. The instrument was set to 25 °C and 1 nm samplings. An average spectrum was calculated from three scans.

For determining the autoxidation rate, about 6.3 µM Mb protein was incubated at 37 °C, in 100 mM sodium phosphate buffer, in the presence of superoxide dismutase and catalase, and 1 mM EDTA for 18 h in a Cary60 spectrophotometer (Agilent, CA, USA). Prior to the measurements, the stored carboxy-Mb solution was buffer exchanged with a Sephadex G25 PD10 column, bubbled with oxygen and buffer exchanged again with a Sephadex G25 PD10 column, using air-equilibrated buffer. Absorbance measurements were conducted every 30 min in the range 350–700 nm. After the measurements were finished, an excess of potassium ferricyanide was added to complete the conversion to met-Mb. The autoxidation rate was calculated from the decrease in absorbance at 581 nm. A least squares algorithm (excel solver, non-linear GRG) was used to fit the data to an exponential equation. The absorbance after ferricyanide addition was used as offset during the curve fitting.

In order to investigate thermal unfolding and aggregation, the purified oxy-Mb protein was first prepared from the Mb stock solution by two consecutive buffer changes into air equilibrated phosphate buffered saline (PBS) using Sephadex G25 PD10 columns. Absorbance spectroscopy was used to confirm conversion to the oxy form. The samples were tested, in triplicate, in either the oxy-Mb form or after bubbling with CO. The samples were monitored by fluorescence detection at 330 nm and 350 nm and by light extinction measurements, over a temperature range from 20 °C to 95 °C using a Prometheus NT.48 instrument (Nano Temper Technologies, Munich, Germany). A temperature increase of 1 °C/min and 90% excitation power were used. PR.therm Control v.2.04 was used for determination of inflection points and onset values for each replicate. An initial test of the heat stability of carboxy-Mb in the protein extract was also done to help identify the band in SDS-PAGE. For that test, the sample was heated at 55 °C for 5 min under CO.

The stability of the met form of the purified Mb was estimated through denaturation by guanidine-HCl (Gu-HCl) and low pH. Oxy-Mb was first prepared by removing CO with two steps of buffer change using air equilibrated buffers and Sephadex G25 PD10 columns (GE Healthcare), followed by generation of met-Mb by the addition of a ferricyanide solution to a final concentration of 1.5x molar excess relative Mb. The sample was then buffer changed using Sephadex G25 PD10 columns (GE Healthcare) into 100 mM sodium phosphate buffer pH 7, 100 mM KCl and incubated in the Gu-HCl solution with final concentrations of 0–3 M at room temperature for approximatively 24 h. The samples were then measured at 409 nm using a Multiskan GO plate reader (Thermo Fisher Scientific). For the pH stability test, the met-Mb form was prepared as above, but buffer changed to Millipore water, and then diluted 1:10 in the appropriate buffer along with 100 mM KCl (100 mM sodium phosphate buffer at pH 7, 6, 3 and 2 or sodium acetate buffer at pH 5 and 4). Following ~30 min incubation at room temperature, the sample absorbance spectra were measured using a Multiskan GO Spectrophotometer (Thermo Fisher Scientific).

## Results

### Vector confirmation and transient production

**Figure 1:**
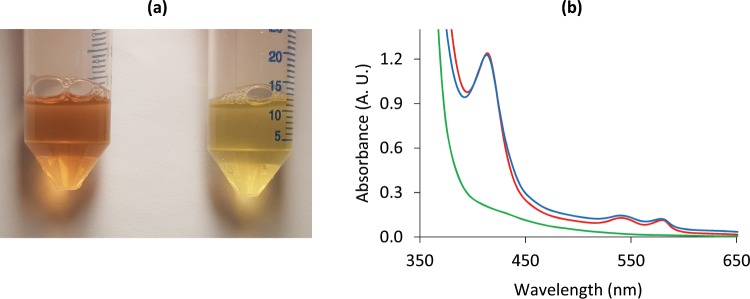
Effects of Mb expression on the color (**a**) and absorbance **(b)** of *N*. *benthamiana* leaf extracts. **(a)** Image of protein extracts from agroinfiltrated leaves expressing myoglobin (left) and protein extracts from untreated leaves (right). (**b**) Absorbance spectra of clarified and filtered extracts: red: infiltrated, “cytosol accumulated” myoglobin construct, blue: infiltrated, “chloroplast targeted” myoglobin construct, green: Untreated *N*. *benthamiana*.

**Figure 2:**
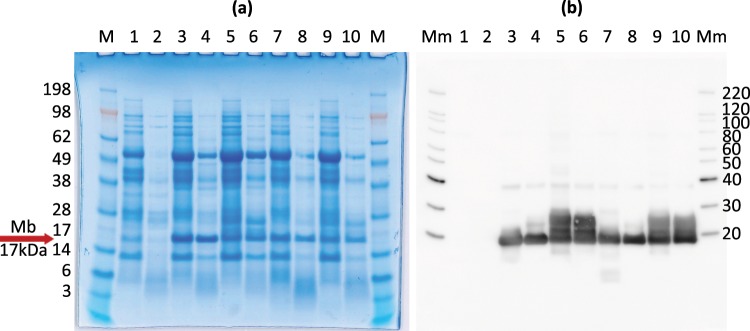
SDS-PAGE (**a)** and Western blot **(b)** analyses of *N*. *benthamiana* leaf extracts. Red arrows indicate putative Mb bands. Each sample was loaded first as the raw extract, then as heat treated extract following a short heat step (55 °C, 5 min, CO bubbled). Lanes 1–2: untreated sample; 3–4: agroinfiltrated, cytosol accumulated Mb; 5–6: agroinfiltrated, chloroplast targeted Mb; 7–8: agrosprayed, cytosol accumulated Mb; 9–10: agrosprayed, chloroplast targeted Mb. M: Seeblueplus2. Mm: MagicMark XP.

The target sequences were successfully inserted into the pJL-TRBO vector, which was confirmed by sequencing. Thus, two transformation vectors were generated. Transformations of the pJL-TRBO-Mb vectors into *E*. *coli* and *Agrobacterium* were verified by colony PCR. The successful expression of the Mb protein was detected in both agroinfiltrated and agrosprayed leaves of *N*. *benthamiana*. The presence of Mb protein in the clarified extracts was visually apparent from the color of the extracts (Fig. 1a), and confirmed by absorbance spectroscopy (Fig. 1b). The absorbance spectra of the extracts (Fig. 1b) indicated a similar level of expression for the cytosol accumulated and the chloroplast targeted Mb. SDS-PAGE and Western blot analyses indicated that the expressed Mb protein was of the expected size (17 kDa) and displayed the expected epitope for antibody binding and thermal stability. The chloroplast targeted Mb appeared to display size heterogeneity though (Fig. 2). Further work was therefore focused on the cytosol accumulated Mb protein.

### Purification and yield

**Figure 3:**
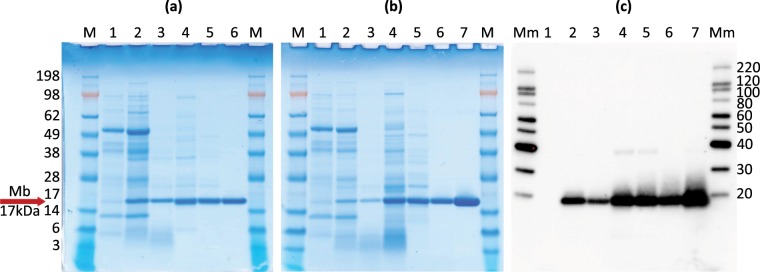
SDS-PAGE and Western blot analyses. **(a**) SDS-PAGE of protein extracts from *N*. *benthamiana* leaves showing the expressed Mb following agroinfiltration and its purity after each successive step of purification. Lane 1: control (extract of untreated leaves); Lane 2: raw extract; Lane 3: heated extract, Lane 4: concentrated (ca. 15x) and buffer exchanged extract, Lane 5: ammonium sulfate fractionated and buffer changed extract, Lane 6: anion exchange purified Mb. Note: 6 µl sample was loaded in lanes 1–3 and ~1 µl sample was loaded in lanes 4–6. **(b**,**c)** SDS-PAGE and Western blot of protein extracts from *N*. *benthamiana* leaves showing the expressed Mb following agrospray and its purity after each successive step of purification. Lane 1: control (extract of untreated leaves), Lane 2: raw extract, Lane 3: heated extract, Lane 4: concentrated extract (ca. 28x), Lane 5: ammonium sulfate fractionated and buffer changed extract, Lane 6: anion exchange purified Mb, Lane 7: anion exchange purified Mb (high conc. ~6 ng loaded). Note: 6 µl sample was loaded in lanes 1–3 and 2 µl in lanes 4–6. For whole figure: the total sample volume was kept roughly constant following the concentration step (lanes 4–6) to facilitate yield estimation. M: Seeblueplus2. Mm: MagicMark XP. (Full size gels are available in Supplementary Fig. S1).

Purification of the Mb protein was achieved by initially removing the bulk of the non-target compounds with heat, diafiltration and ammonium sulfate fractionation. These methods were effective in removing the majority of the non-target compounds in the extracts with a good yield of the Mb protein. The final anion-exchange chromatography step left a highly purified Mb protein. SDS-PAGE and Western blot analysis were used to evaluate the purity and yield of the protein after each step of purification (Fig. 3). The chromatogram also showed the effectiveness of the purification (Supplementary Fig. S2). A possible dimer band was detected on the Western blot (Fig. 3c), but was almost entirely removed after anion exchange chromatography. The height of the absorbance maximum at ~540 nm in relation to the absorbance maximum around 280 nm, could also be used as an estimate of the protein purity. Relevant absorbance ratios were 0.246 (541 nm/277 nm) and 0.376 (541 nm/273 nm) for the sprayed samples before and after chromatographic purification, respectively. Prior to the chromatography purification, the protein purity was thus approximatively 65% of the final quality. For the agroinfiltrated sample, a ratio of 0.389 (540 nm/274 nm) was achieved after chromatographic purification (Supplementary Fig. S3). A similar ratio calculated from the corresponding extinction coefficients reported by Antonini and Brunori^4^ for sperm whale Mb following purification of Mb was 0.373 (542 nm/275 nm). Our results indicate that the plant produced Mb was of high purity following chromatography.

The yield of chromatography purified Mb protein following agroinfiltration was estimated as ~210 mg/kg fresh weight leaf tissue, based on the carboxy-Mb absorbance spectra. The yield of purified Mb protein following agrospray was about 60–80 mg/kg and the variation is likely partly due to variation in the heating time, which was more strictly limited to ten minutes for the final agrosprayed batch (~80 mg/kg, Fig. 3) and the agroinfiltrated material. A rough estimation of the Mb protein in the unpurified sprayed sample extract was ~120 mg/kg, based on subtraction of the estimated absorbance background, indicating a percentage yield over the purification of around ~50–70%.

### Protein analysis

**Figure 4:**
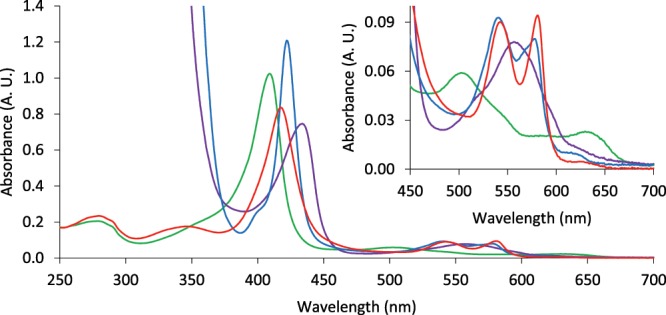
UV-Vis absorbance spectra of Mb produced in the leaves of *N*. *benthamiana*, measured at 250–700 nm, with an enlarged section at 450–700 nm. oxy-Mb (red), carboxy-Mb (blue), deoxy-Mb (purple), met-Mb (green). Sodium dithionite absorbance dominates the spectrum below approximatively 390 nm for the displayed carboxy-Mb and deoxy-Mb spectra. (See Supplementary Table S1 for absorbance values for selected wavelengths).

The formation of different ligand-forms of Mb, namely oxy-Mb, carboxy-Mb, deoxy-Mb and met-Mb were detected by UV-Vis absorbance spectrometry, following exposure to the appropriate conditions and procedures, demonstrating the protein’s ability to bind and release its oxygen and CO ligands (Fig. 4) (Supplementary Table S1). The plant produced Mb generated absorption spectra that correspond well, in terms of peak position and relative peak height, with the data presented by Antonini and Brunori^4^ and Hardman *et al*.^33^ for corresponding ligand forms of sperm whale Mb and with the data presented for the native human carboxy-Mb at 528–586 nm by de Duve^32^.

**Figure 5:**
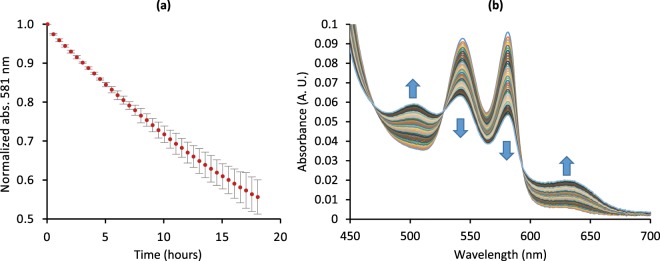
Autoxidation test. (**a**) Autoxidation of Mb produced in *N*. *benthamiana* as followed by the decrease in normalized absorbance at 581 nm (average from three replicates, 95% confidence interval, showing the curve deviation). (**b**) Absorbance spectra (average of three samples) in 30 min intervals over 18 h. Arrows indicate overall trend over time.

**Figure 6:**
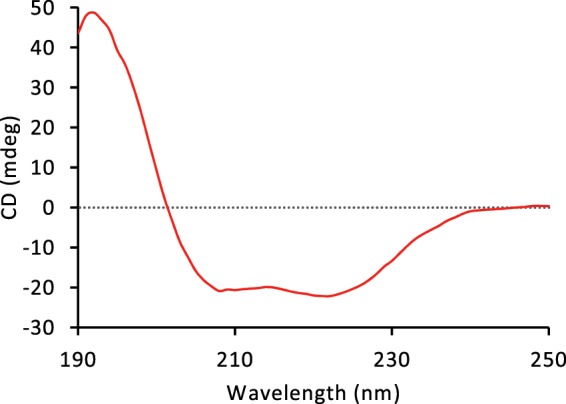
Circular dichroism spectra of purified Mb protein produced in the leaves of *N*. *benthamiana*.

LC-ESI-MS analysis of the purified Mb protein showed a single prominent peak with a mass of 17052.35 Da, which indicates successful removal of the initiator methionine, given the expected theoretical apo-Mb mass of 17052.61 Da, as calculated by the ExPASy ProtParam tool^34^, and the experimentally determined value 17053.0 Da reported by Deterding *et al*.^35^. This also indicates that no unexpected posttranslational modifications were present in the purified Mb protein. An approximate autoxidation rate of 0.046 ± 0.004 h^−1^ (SD, n = 3) was recorded (Fig. 5). The recorded CD spectrum (Fig. 6) was typical of an alpha helix dominated protein, a characteristic property of Mb^36^.

**Figure 7:**
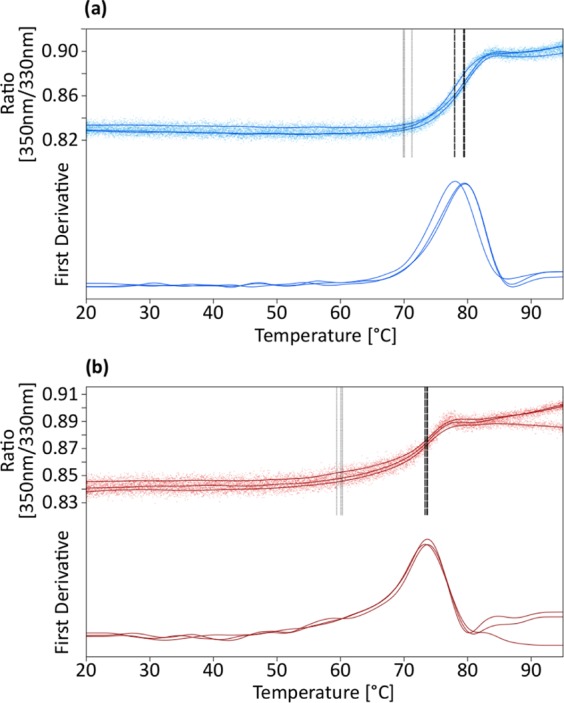
Temperature stability analysis (triplicates) by differential scanning fluorimetry. **(a)** oxy-Mb. **(b)** carboxy-Mb. The measured fluorescence ratio data and its corresponding first derivatives are displayed for each replicate. Both smoothed curves and data points are shown in the figure. Vertical dotted lines indicate the detected onset (gray) and melting temperatures (black) in the top graphs.

Temperature stability data, as determined by differential scanning fluorimetry (DSF), for oxy-Mb and carboxy-Mb samples is presented in Fig. 7. The detected inflection point (Tm) of the CO bubbled sample was determined as 78.9 ± 2.2 °C (95% confidence interval, n = 3, for this and the following values) with an onset of 70.4 ± 1.7 °C, while the oxy-Mb sample showed a corresponding Tm of 73.5 ± 1.1 °C and onset at 59.9 ± 0.5 °C. The fluorescence data appeared to indicate a more gradual response to temperature for the oxy-Mb sample than for the carboxy-Mb, which is not surprising as autoxidation and resulting early unfolding and heme loss are likely to occur under these conditions. Light scattering data was also collected further supporting a more complex degradation process for the oxy-Mb sample during the test (Supplementary Fig. S4).

**Figure 8:**
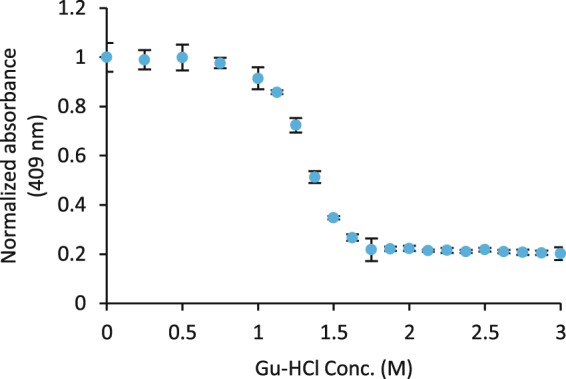
Change in absorption (409 nm) due to denaturation of plant produced Mb by Gu-HCl (95% conf. interval, n = 3).

Gu-HCl unfolding tests of met-Mb showed an apparent denaturation midpoint at approximatively 1.3 M Gu-HCl. (Fig. 8). An estimation of ΔG_0_ from the denaturation curve, performed essentially as described by Sykes *et al*.^37^, and using the averages of the 0 M Gu-HCl and 3 M Gu-HCl absorbance measurements, as initial and final values, yielded a value of about 27.5 ± 0.5 kJ/mol (Standard error of intercept, n = 9) (Supplementary Fig. S5). This value appeared to vary somewhat between experiments performed under comparable conditions. The pH stability test indicated that the met-Mb heme binding was disrupted between pH 4 and 5, as expected^38^ (Supplementary Fig. S6).

## Discussion

The present study has shown that the plant expressed human Mb protein had similar physical properties to other mammalian Mb proteins and displayed its functional activity, through binding and dissociation of its ligands. The positions of the α- and β-peaks of the carboxy-Mb spectra are largely in agreement with those reported by de Duve^32^ for human Mb. Moreover, the detected spectra are also in good agreement with the absorption spectra of sperm whale oxy-, deoxy-, carboxy- and met-Mb^33^, while the peak positions and relative extinction coefficients for the peaks correspond well with previously reported data^4,33^. The minor peak around 620 nm in the carboxy- and oxy-spectra might indicate the presence of a small fraction of non-functional Mb, such as met- or sulf-Mb or other degradation products. The close similarity of the spectra to previously reported data for human and sperm whale Mb indicates that the folding of the human Mb protein and incorporation of the heme in the plant cytoplasm were correctly performed. The CD data was also consistent with correctly folded Mb.

The plants appeared to be capable of supplying a large amount of heme for holo-Mb production in the study, even with the short timeframe of production following agroinfiltration, indicating either the presence of a large pool of heme, or a rapid increase in the heme biosynthesis following the increased cellular demand for the cofactor. The chloroplast targeted construct appeared to produce a similar concentration of Mb as the non-targeted and cytosol accumulated Mb construct. This might indicate that the levels of accessible heme were similar in the two compartments, or that the heme access was not a determining factor for the Mb yield, however, as the cellular localization of the produced Mb protein and of the heme incorporation event were not determined, no final conclusions could be drawn for this.

The detected autoxidation rate (0.046 ± 0.004 h^−1^ (SD, n = 3)) of the plant produced Mb in the presence of superoxide dismutase and catalase was similar to that (0.055 ± 0.005 h^−1^ (SD, n ≥ 3)) of sperm whale Mb reported by Brantley *et al*.^39^. Differential scanning fluorimetry showed that the Mb protein was stable at relatively high temperatures, and was stabilized effectively by CO binding. The CO binding probably stabilized the Mb protein in part by preventing its autoxidation, as has been noted by other authors for carboxy-Mb^33^ and carboxy-Hb^40^. The Gu-HCl experiment also showed the stability of the ferric protein, even though the level was somewhat lower than that reported for horse heart met-Mb with similar methods^37^.

Overall, the analyzed properties of the plant produced Mb were similar to the human Mb protein and to other mammalian Mb proteins for properties where no data for human Mb was found for comparison.

The pTRBO viral vector used for transient protein expression in this study relies on the self-replication and cell-to-cell movement ability of a viral element, following entry into the plant cell mediated by the *Agrobacterium*^29^. This allows distribution of myoglobin expression throughout an infected leaf given enough time, even with a small initial amount of *Agrobacterium*. In this study, two *Agrobacterium* delivery methods were used, namely agroinfiltration, using a high concentration of *Agrobacterium* suspension and agrospray, using diluted *Agrobacterium* suspension. Although higher levels of Mb production were observed in the agroinfiltrated leaves, the agrospray method could be more advantageous as it reduces the labor input, and would thus facilitate scale up and avoid induced stress responses, which may complicate the protein purification work. Agrospray with the viral vectors has previously been shown to be an effective tool for transient protein expression^41,42^. Since the agrospray method, as used here, relied to a higher degree on the replication of the viral vector, with a smaller amount of *Agrobacterium* expected to enter the plant leaves, it could perhaps reduce the endotoxin load compared to the agroinfiltration procedure. As the lack of immunogenic lipopolysaccharides is a major advantage of plant expression systems compared to bacterial, where endotoxins are a major concern^18^, minimizing the endotoxin load could be an important factor, when designing a transient expression protocol for protein production for medical and nutritional applications.

An effective and inexpensive protocol for protein isolation and purification is critical for large-scale production of proteins intended for nutritional applications. Similarly, it would be very important for production of pharmaceuticals, and in particular oxygen pharmaceuticals, due to the large quantities involved^18^, although the purity requirements are higher for such uses. In this study, a relatively high degree of purity could be achieved with relatively simple, low-cost methods, in which the heat treatment and ammonium sulfate precipitation removed the bulk of the native tobacco proteins, while diafiltration removed small molecule contaminants. Heating protocols have previously been shown to be effective for removing native tobacco proteins during recombinant protein purification^43,44^ and ammonium sulfate has long been known to be effective for Mb isolation^4,32^. Following these initial purification procedures, a high degree of purity, required for reliable characterization and pharmaceutical applications, could be achieved by a single anion exchange chromatography step, a method often combined with ammonium sulfate fractionation for Mb purification from meat^45,46^.

In this study, we have shown that functional Mb protein, with properties similar to native Mbs could be produced in the green leaf tissues of *N*. *benthamiana*, without externally supplied heme, and isolated efficiently using comparatively simple and inexpensive methods. Production of functional myoglobin in tobacco helps to show the feasibility of using the plant system for producing heme-proteins that could be suitable for pharmaceutical applications. The results also demonstrate the possibility of using plant produced heme-proteins for iron nutrition or other “meat substitute” purposes. In this article, the human Mb gene was used to investigate the plant production system for protein production, but Mbs from other species, more appealing to consumption, could most likely be produced with this approach. For estimating the economic feasibility of production of Mb as a food additive using recombinant techniques, further careful evaluation of costs for production and down-stream processing, taking economies of scale into account, would be needed. Further improvements of yield and processing methodology might still be required, though. Additionally, the development of high expression transgenic plants and the use of edible crops for the production of heme-proteins could also be interesting options for further inquiry. Although the total heme content in the plant tissues was not determined in this study, the amount of heme protein in the soluble fraction appeared to be increased substantially, as shown in Fig. 1, which might indicate an increase in total heme level, with potential implications for future heme-iron biofortification of food crops. The Mb heme iron concentrations, calculated based on the yield of purified Mb in this study, (~0.7 mg heme iron/kg for agroinfiltration, ~0.24 mg heme iron/kg for agrospray) are lower than the values reported for chicken, pork and beef meats (~2–10 mg heme iron/kg)^47^. Assuming some losses in purification however, the Mb heme concentration in the agroinfiltrated tobacco tissues might not be far from chicken or pork, at the lower end of that range.

In conclusion, this study has shown that the human Mb protein could be successfully expressed in *N*. *benthamiana* leaves by transient expression using a viral vector delivered by *Agrobacterium*. Both the agroinfiltration and the more scalable agrospray methods were effective, but the agroinfiltration method resulted in higher levels of Mb production. The produced Mb protein could be purified to a relatively high degree of purity using comparatively low cost methods. The purified Mb protein was shown to be functional, in terms of ligand binding, and had properties similar to native Mb proteins. The results indicate that plants could be a useful production platform for Mbs or other types of heme-proteins for potential future nutritional or medical applications.

## Supplementary information

Supplementary information.
