# Supplementary material for: Plant based production of myoglobin - a novel source of the muscle heme-protein
Source: Sci Rep. 2020 Jan 22;10:920. doi: 10.1038/s41598-020-57565-y (PMC6976567; doi:10.1038/s41598-020-57565-y)
Supplement: Supplementary file 1 — Supplementary information. [file 41598_2020_57565_MOESM1_ESM.pdf]

Supplementary information: Plant based production of myoglobin - a novel source of the muscle heme-protein

Magnus L. R. Carlsson[1], Selvaraju Kanagarajan[1], Leif Bülow[2], Li-Hua Zhu[1]*

[1]Department of Plant Breeding, Swedish University of Agricultural Sciences, Box 101, 230 53 Alnarp, Sweden

[2]Division of Pure and Applied Biochemistry, Department of Chemistry, Lund University, Box 124, 221 00 Lund, Sweden

*Corresponding author, E-mail: Li-Hua.Zhu@slu.se

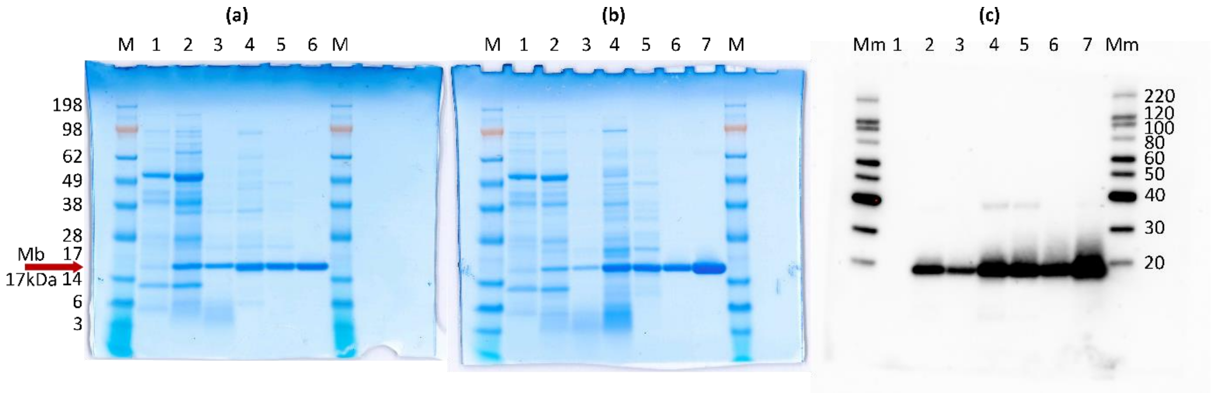

Supplementary figure S1. Full size gel and WB images from "figure 3" – **(a)**: SDS-PAGE of protein extracts (3:1 parts buffer to solid) from *N. benthamiana* leaves showing the expressed Mb following agroinfiltration and its purity after each successive step of purification. Lane 1: control (extract of untreated leaves); Lane 2: raw extract; Lane 3: heated extract, Lane 4: concentrated (ca. 15x) and buffer exchanged extract, Lane 5: ammonium sulfate fractionated and buffer changed extract, Lane 6: anion exchange purified Mb. Note: 6 µl sample was loaded in lanes 1-3 and ~1 µl sample was loaded in lanes 4-6. **(b)** and **(c)**: SDS-PAGE and Western blot of protein extracts (3:1 parts buffer to solid) from *N. benthamiana* leaves showing the expressed Mb following agrospray and its purity after each successive step of purification. Lane 1: control (extract of untreated leaves), Lane 2: raw extract, Lane 3: heated extract, Lane 4: concentrated extract (ca. 28x), Lane 5: ammonium sulfate fractionated and buffer changed extract, Lane 6: anion exchange purified Mb, Lane 7: anion exchange purified Mb (high conc. ~6ng loaded). Note: 6 µl sample was loaded in lanes 1-3 and 2 µl in lanes 4-6. For whole figure: total sample volume was kept roughly constant following the concentration step (lanes 4-6), to facilitate yield estimation. M: Seeblueplus2. Mm: MagicMark XP.

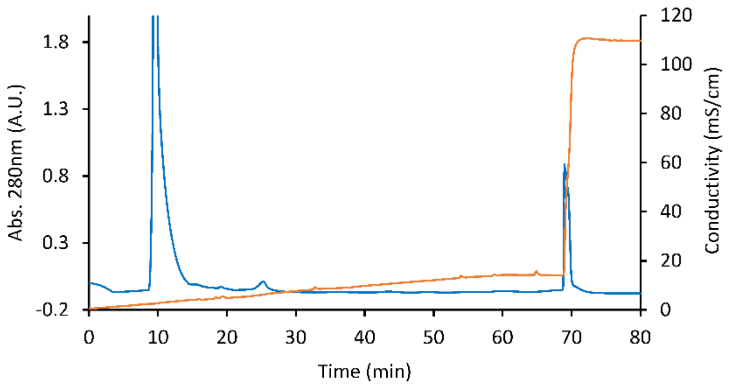

Supplementary figure S2. Chromatogram showing the elusion step following binding of the agroinfiltrated leaf extract to Q-sepharose (3x5 ml Hitrap HP) with the main myoglobin elution peak at app. 10-15 min. The elusion was performed with a 0-100 mM NaCl gradient at 5ml/min, followed by a 1 M NaCl wash step at ca. 70 min. Blue graph: detected absorbance (left axis). Orange graph: detected conductivity (right axis).

Supplementary table S1. Selected absorbance values from spectra presented in figure 4, corresponding to the main observed peaks, same start concentration for tested samples, but some variation possible due to evaporation during gas bubbling and added dithionite and ferricyanide.

Sample	Wavelength (nm)	Absorbance (A.U.)
Oxy-Mb		
	280	0.234
	347	0.1757
	418	0.8347
	542/543	0.0901
	581	0.0942
Carboxy-Mb (no dithionite)		
	272	0.2359
	344	0.1752
	422	1.1974
	541	0.0906
	578	0.0777
Carboxy-Mb (dithionite)		
	422	1.208
	541	0.0928
	578	0.0799
Deoxy-Mb (dithionite)		
	434	0.7453
	556/557	0.0779
Met-Mb (ferricyanide)		
	279	0.2052
	409	1.024
	502	0.0591
	630	0.0231

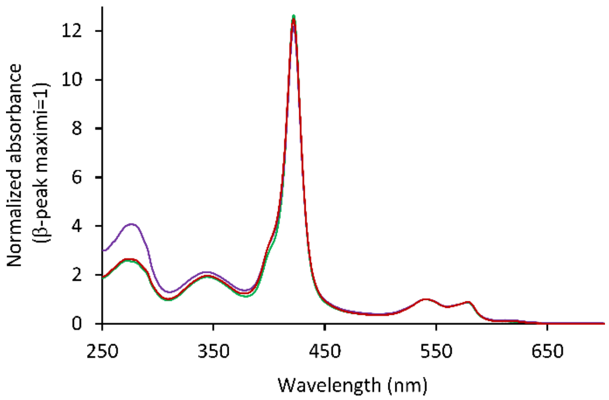

Supplementary figure S3. UV-Vis absorbance spectra used for purity assessment of produced Mb, samples were bubbled with CO before measurement. Purple: before chromatography (agrospray); Red: after chromatography (agrospray). Green: after chromatography (agroinfiltration). The Soret peak (~422 nm) was most likely outside the linear range of the detector and the height shown here may not be reliable.

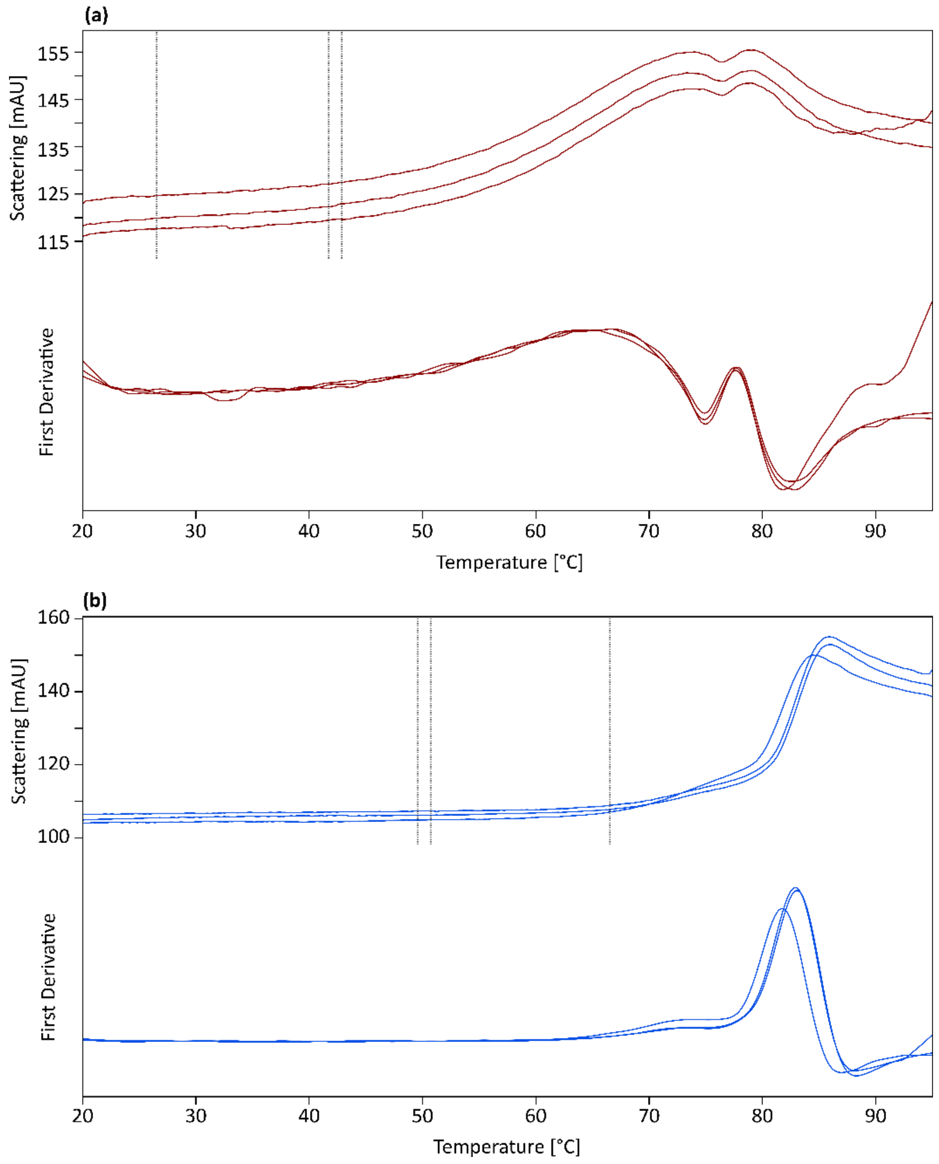

Supplementary figure S4. Temperature stability analysis (triplicates) by light scattering. **(a)**: oxy-Mb. **(b)**: carboxy-Mb. Light scattering data (upper graphs) and its corresponding first derivatives (lower graphs) are displayed for each replicate. Detected onset temperatures displayed as vertical lines in gray. Note: The wavelength for the light scattering measurement is not disclosed by the instrument manufacturer hence the result may reflect myoglobin absorbance change, for example due to autoxidation, as well as light scattering.

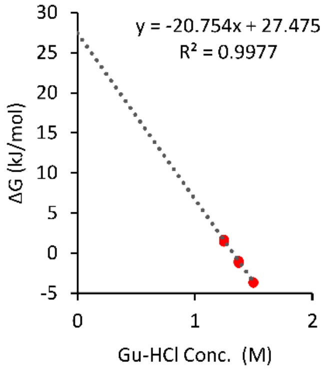

Supplementary figure S5. Regression analysis for determination of ΔG_0 from gu-HCL denaturation curve. (9 datapoints at 3 denaturant concentrations used for fit, partially overlapping).

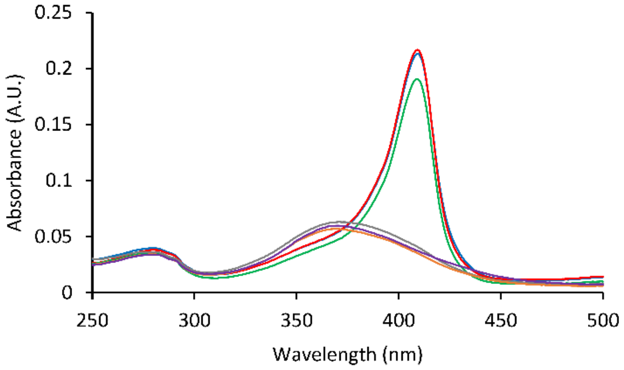

Supplementary figure S6. pH stability test of plant produced Mb in met-form using UV-Vis spectroscopy. Blue: pH 7, red: pH 6, green: pH 5, grey: pH 4, orange: pH 3, purple: pH 2.
